# Modulating Neuroinflammation as a Prospective Therapeutic Target in Alzheimer’s Disease

**DOI:** 10.3390/cells14030168

**Published:** 2025-01-22

**Authors:** Eunshil Lee, Yongmin Chang

**Affiliations:** 1 Institute of Biomedical Engineering Research, Kyungpook National University, Daegu 41944, Republic of Korea; leees82@naver.com; 2 Department of Molecular Medicine, School of Medicine, Kyungpook National University, Daegu 41944, Republic of Korea; 3 Department of Radiology, Kyungpook National University Hospital, Daegu 41944, Republic of Korea

**Keywords:** neuroinflammation, microglia, receptors, pathways, Alzheimer’s disease

## Abstract

The recent approval of lecanemab highlights that the amyloid beta (Aβ) protein is an important pathological target in Alzheimer’s disease (AD) and further emphasizes the significance of neuroinflammatory pathways in regulating Aβ accumulation. Indeed, Aβ accumulation triggers microglia activation, which are key mediators in neuroinflammation. The inflammatory responses in this process can lead to neuronal damage and functional decline. Microglia secrete proinflammatory cytokines that accelerate neuronal death and release anti-inflammatory cytokines and growth factors contributing to neuronal recovery and protection. Thus, microglia play a dual role in neurodegeneration and neuroprotection, complicating their function in AD. Therefore, elucidating the complex interactions between Aβ protein, microglia, and neuroinflammation is essential for developing new strategies for treating AD. This review investigates the receptors and pathways involved in activating microglia and aims to enhance understanding of how these processes impact neuroinflammation in AD, as well as how they can be regulated. This review also analyzed studies reported in the existing literature and ongoing clinical trials. Overall, these studies will contribute to understanding the regulatory mechanisms of neuroinflammation and developing new therapies that can slow the pathological progression of AD.

## 1. Alzheimer’s Disease

Alzheimer’s disease (AD) is a progressive neurological condition that manifests through cognitive decline, loss of episodic and working memory, language difficulties, and behavioral changes [1]. AD represents the most common form of dementia worldwide, accounting for at least two-thirds of cases in individuals aged 65 and older [1]. AD places a significant burden on patients and their families, impacting social, psychological, physical, and economic aspects of life. Additionally, with the increase in global life expectancy, the number of patients with AD is expected to rise dramatically, thus amplifying the need for effective treatment and care strategies.

AD is a multifactorial condition associated with various risk factors, including genetic mutations in proteins such as apolipoprotein E (APOE) and triggering receptors expressed on myeloid cells 2 (TREM2), alongside age, cardiovascular disease, obesity, and diabetes. The histopathological changes in AD can primarily be summarized into two key features [1,2,3,4,5]. Firstly, the accumulation of amyloid plaques is characteristic of AD. These plaques mainly consist of amyloid beta (Aβ) peptides and disrupt synaptic function, making their considerations essential in the neuropathological diagnosis of AD. Amyloid plaques hinder neuron communication and affect memory and cognitive functions [6,7]. Secondly, an increase is observed in neurofibrillary tangles (NFTs). These NFTs are formed by hyperphosphorylated tau proteins and strongly correlate with AD severity. The accumulation of NFTs severely impacts neuronal function, leading to cell death and a decline in cognitive functions [7,8,9]. Moreover, these histopathological changes interact and contribute to AD progression. Amyloid and tau pathologies primarily appear in the early and middle stages of AD, leading to synaptic degeneration and loss, which result in neuronal death in the cerebral cortex and hippocampus [1]. Additionally, the immune responses of microglia and other immune cells can exacerbate amyloid and tau pathologies, which have emerged as significant contributors to the disease’s pathology [10,11,12].

Despite advancements in the understanding of AD, there are presently no treatments available that can prevent or reverse disease progression. However, known AD treatments include the cholinesterase inhibitor tacrine, which was first approved in 1993, as well as other medications, such as donepezil and galantamine. The NMDA receptor antagonist memantine was approved in 2003. However, cholinesterase inhibitors and NMDA receptor antagonists are primarily classified as symptomatic treatments, as they mainly temporarily relieve symptoms or control neuropsychiatric symptoms, distinguishing them from disease-modifying treatments (DMTs) that affect the underlying biological AD pathology. Before the approval of lecanemab, available AD treatments primarily alleviated symptoms temporarily, prompting many pharmaceutical companies to focus on developing DMTs that target the underlying causes of the disease. As a result, the Aβ-targeting immunotherapy lecanemab received traditional approval from the U.S. FDA in July 2023 for patients with early-stage AD. This marked the first treatment specifically targeting the underlying pathological mechanisms of AD, representing a significant advancement in DMTs and offering great potential for slowing disease progression in patients. Donanemab subsequently received FDA approval in July 2024.

As of 2024, various DMT candidates with different CADRO targets are in clinical trials [13]. Notably, according to reports from 2024, the mechanisms most studied in clinical trials based on CADRO relate to neurotransmitters, inflammation, and amyloid targets. These mechanisms present various approaches to address the complex pathological processes of AD and are expected to contribute to the diversification of future treatment strategies.

In particular, neuroinflammation is recognized as a significant pathological factor in AD, with the abnormal activation of microglia and its association with disease progression becoming increasingly emphasized. In a recent comprehensive review [14,15], neuroinflammation was introduced as a pathological hallmark of AD, further highlighting the critical role of microglial activation in shaping the pathological landscape and driving disease progression. During the progression of AD, microglia are abnormally activated in response to external signals such as protein aggregates, such as Aβ and tau, or cellular damage signals, and may transition into various states, including disease-associated microglia (DAMs) and microglial neurodegenerative phenotype (MGnD). Additionally, genetic mutations in microglia can lead to changes in their function, potentially accelerating AD progression. These changes regulate cytokine production, phagocytosis, and reactive oxygen species (ROS) production and influence synaptic function, promoting neuronal damage and cell death. Such neuroinflammatory responses have become a pathological hallmark of AD, driving neurodegeneration through increased inflammatory responses, oxidative stress, synaptic elimination, cellular damage, and amyloid and tau accumulation. The fact that neuroinflammation represents the second most common drug target in clinical trials for AD treatments suggests that inflammatory responses may play a significant role in promoting the major pathological processes of AD [13]. Indeed, this theory extends beyond treatment strategies limited to amyloid or tau accumulation, highlighting that neuroinflammation is recognized as a critical contributing factor in AD pathology; thus, various therapeutic approaches that target this aspect are necessary. In this context, the role of neuroinflammation in the pathology of AD is supported by several studies. Indeed, studies have confirmed the presence of complement proteins (C1q, C3, C4, etc.) within amyloid plaques in AD patients, suggesting immune responses may be involved in plaque formation [16,17]. Additionally, the increase in proinflammatory and anti-inflammatory cytokines in the brains and cerebrospinal fluid (CSF) of AD patients indicates that these inflammatory responses are significant elements in AD pathology [18,19,20]. Moreover, although clinical trials have yet to yield significant results, research using AD mouse models has demonstrated that non-steroidal anti-inflammatory drugs may alleviate inflammatory responses and potentially reduce the risk of developing AD [21,22,23]. Therefore, neuroinflammation has become an important aspect of studying AD pathogenesis [24,25,26,27,28].

A significant number of scientific studies suggest that neuroinflammation could be targeted as a novel therapeutic objective in developing treatment strategies for AD. These studies provide evidence that modulating inflammatory mechanisms may help improve patient outcomes. Thus, future research needs to develop a deeper understanding of the role of neuroinflammation and acquire effective treatments based on this understanding.

## 2. Neuroinflammation

Recent studies on the pathogenesis of AD present various perspectives on the role of neuroinflammation. Many studies describe neuroinflammation as a secondary phenomenon that arises during disease progression, noting that even after the accumulation of Aβ, the inflammatory response is critical in forming a feedback loop that exacerbates neuronal damage and pathological changes [29]. However, other reviews suggest that neuroinflammatory processes may begin before the significant accumulation of key pathological features such as Aβ and tau and that these processes could play a crucial role in the onset of AD [30,31]. Additionally, according to the study by Spangenberg and colleagues, microglia play an important role in the early stages of Aβ plaque formation, and the removal of microglia significantly reduced Aβ plaque seeding [32]. These results suggest that microglia are actively involved in the crucial stages preceding Aβ plaque formation. However, much conflicting data exist, and the roles of microglia may vary depending on factors such as the stage of disease progression, the experimental model used, genetic mutations, and the activation state of microglia. Hence, understanding these factors requires further research, which will contribute to unraveling the complex role of microglia in AD.

The activation of immune cells, such as microglia and astrocytes, in the nervous system triggers neuroinflammation. Indeed, these cells play essential roles in regulating immune responses in the central nervous system (CNS) and in supporting the survival and function of neurons. Microglia and astrocytes interact through the secretion of molecules such as growth factors, cytokines, and chemokines, which play important roles in regulating inflammatory responses, activation, and phagocytosis [33]. Cytokines, prostaglandins, and various molecules secreted by microglia induce inflammatory responses and promote the proliferation of astrocytes [34,35]. In particular, microglia activation plays a crucial role in regulating the immune functions of astrocytes and promoting astrogliosis [36]. Additionally, TGFβ and chemokines, among other factors secreted by astrocytes, have been reported to affect the activation and motility of microglia [37,38,39,40]. These interactions have significant implications for the progression of neuroinflammation and AD.

Microglia and astrocytes interact closely and regulate inflammatory responses; however, this review will specifically examine the receptors and signaling pathways leading to microglia activation. This review will also provide a comprehensive review based on studies reported in the existing literature and ongoing clinical trials, broadening our understanding of the relationship between neuroinflammation and AD.

### 2.1. Microglia

Microglia are resident immune cells widely distributed across the CNS and are essential for immune surveillance, regulating inflammatory responses, and synaptic remodeling [41,42]. Microglia detect damage-associated molecular patterns (DAMPs) released from damaged neurons or abnormal cellular environments and pathogen-associated molecular patterns (PAMPs) derived from external pathogens or environmental toxins [43].

Microglia utilize various receptors, such as Toll-like receptors (TLRs), NOD-like receptors (NLRs), RAGE, purinergic receptors, and others, to detect these signals [44]. After detecting inflammatory signals, microglia secrete proinflammatory cytokines (e.g., IL-1β, TNF-α, and IL-6) and anti-inflammatory cytokines (e.g., IL-4, IL-10, and TGF-β) to regulate the inflammatory response. Microglia also modulate inflammation in the nervous system through processes such as phagocytosis to eliminate damaged cells or pathogens and the secretion of chemokines to recruit immune cells to the inflammation site [45,46]. The ability of microglia to detect and regulate these signals is essential for maintaining the health of the nervous system and inducing appropriate immune responses in the context of damage or disease.

**Figure 1:**
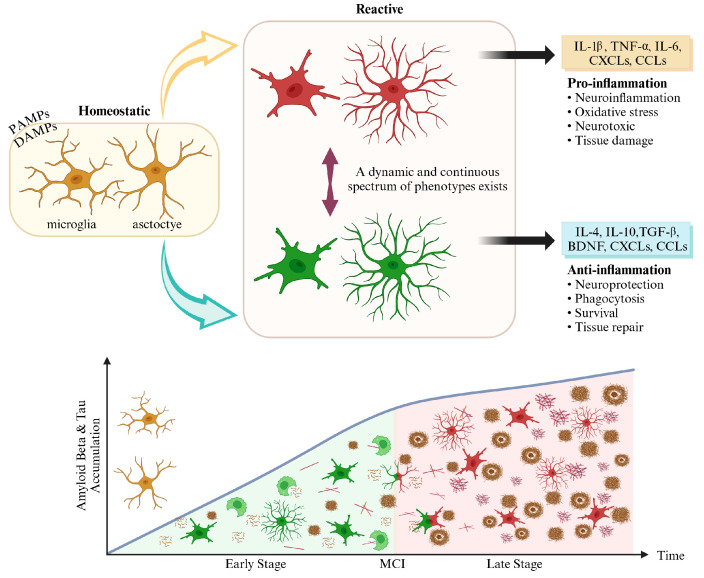
Microglial and astrocytic polarization during neurodegenerative disease progression. Resting microglia respond to PAMPs and DAMPs by polarizing them into proinflammatory or anti-inflammatory microglia. Proinflammatory microglia are associated with the secretion of IL-1β, TNF-α, IL-6, CXCLs, and CCLs, leading to neuroinflammation, oxidative stress, neurotoxicity, and tissue damage. In contrast, anti-inflammatory microglia release IL-4, IL-10, TGF-β, CXCLs, CCLs, and BDNF, promoting neuroprotection, phagocytosis, survival, and tissue repair. Similarly, astrocytes polarize into different reactive states, which can be broadly categorized as neurotoxic or neuroprotective, depending on the specific pathological context. The early activation of microglia is associated with the accumulation of Aβ and tau proteins. Studies evaluating the temporal changes in microglial activation in patients with mild cognitive impairment (MCI) and AD suggest that microglia play a primarily neuroprotective role in the early stages of AD. However, as the transition from the MCI stage to the dementia stage progresses, microglia shift toward a proinflammatory state.

Microglial cells undergo active morphological changes in response to various signals and are traditionally classified into three main phenotypes (Figure 1) [45]. Microglial cells exhibit a radial morphology in the resting state characterized by multiple long, thin, and highly branched processes [46]. Research has demonstrated that in this resting state, microglial cells play a critical role in maintaining homeostasis within the nervous system, actively surveilling and detecting environmental changes, and responding appropriately [47].

Microglia also become activated in the proinflammatory state (M1) and typically undergo morphological changes to an amoeboid shape, characterized by increased cell body size and reduced branching processes [48,49]. In this state, microglia exhibit inflammatory-promoting activities that include inducing the nuclear factor-kappa B (NF-κB) pathway, producing inflammatory cytokines such as TNF-α, IL-1β, IL-6, IL-12, and IL-23, and expressing CD86, MHC class II, and iNOS [50,51].

In the anti-inflammatory state (M2), microglia also resolve inflammation and promote wound healing and tissue recovery. In this state, microglia release anti-inflammatory cytokines, including IL-4, IL-13, IL-10, and TGF-β, along with CD206, BDNF, and Arg1, and exhibit a relatively stable morphology with well-developed processes [50,51]. M2 microglia play a crucial role in maintaining neural health by facilitating the phagocytosis of cellular debris and misfolded proteins [52,53]. These three phenotypes describe how microglia appropriately respond to and regulate various conditions in the nervous system [49]. However, microglia exhibit diverse intermediate phenotypes, and activated microglia can display varying activation states depending on the surrounding environment [46,54,55].

The above-mentioned findings indicate that the existing simplistic classification of microglial phenotypes as mutually exclusive fails to adequately explain the diverse functions and dynamic changes in microglia in physiological and pathological states. Recent studies on microstructural characteristics have identified various new microglial phenotypes, including “bipolar/rod-shaped” and “ameboid” microglia, with functional investigations ongoing across several pathological conditions [46,56,57]. For example, rod-shaped microglia are primarily found during the early activation phase before the disease progresses from early to late stages, and “bipolar/rod-shaped” microglia have been shown to transform into an amoeboid shape upon lipopolysaccharide (LPS) stimulation [58,59]. Notably, “bipolar/rod-shaped” microglia express anti-inflammatory cytokines, such as IL-10 and TGF-β, and are known to play a primarily neuroprotective role during the recovery processes in aged brains and in the context of pathology or disease progression [58]. Thus, understanding the diverse functions and dynamic regulation of microglia is essential for developing therapeutic strategies to mitigate neuroinflammation [60]. A recent comprehensive review [61] mentioned that microglial phenotypes are defined by specific gene expression changes, which promote microglia morphological alterations. Furthermore, these morphological changes were noted to serve as an important indicator for quantifying the activation levels of microglia, with intermediate activation states such as “bipolar” and “rod-shaped” being identified. Additionally, microglia can exist in alternative activation phenotypes such as DAMs, MGnD, lipid droplet-accumulating microglia, and dark microglia. These diverse phenotypes are closely associated with disease progression and perform various roles, including inflammatory responses, neuroprotection, and enhanced phagocytosis. Furthermore, Leng and Edison’s review [62] emphasizes that pathological stimuli strongly influence microglial morphological changes, which can vary depending on the intensity and duration of the pathological environment. Therefore, understanding the dynamic changes in microglia in detail can provide crucial information for therapeutic approaches. This knowledge enables the development of strategies to modulate microglial activation or guide it toward anti-inflammatory characteristics, which could contribute to treating neurodegenerative diseases such as AD.

The research on sex differences in microglia has recently garnered significant attention. Indeed, according to a review by Han [63], microglia exhibit important differences in density, morphology, and immune responses across various brain regions, depending on sex. Specifically, male microglia exhibit a higher prevalence of amoeboid forms during early development and show increased migration ability, inflammatory responses, and immune activation in response to stimuli. In contrast, female microglia show higher expression and activation of M1 markers in aging mice. However, the exact mechanisms through which these differences impact brain health and disease remain unclear. The sex differences in microglial activation likely contribute to significant variations in immune responses, which could influence the risk of sex-specific neurological diseases.

Reactive microglia play a crucial role in AD. Reactive microglia around senile plaques are concentrated at the core of Aβ deposits, acting as vigilant monitors of the brain by inducing inflammatory responses or actively removing Aβ fragments through key phagocytic activities [64,65]. However, excessive or uncontrolled activation causes an inflammatory phenotype, which may provoke neuroinflammatory responses and exacerbate neuronal damage associated with the Aβ and tau pathology, thereby accelerating disease progression [64,66,67,68]. Numerous studies have focused on the inflammatory functions of microglia in relation to AD. Meanwhile, recent genome-wide association studies (GWASs) have identified proteins associated with microglial function and inflammation, such as TREM2, CD33, CR1, ABCA7, SHIP1, and APOE, which are linked to AD risk [69,70]. A unique microglia phenotype, characterized by a hybrid activation state known as DAMs, was also observed in AD patients. This state influences microglial function, with DAMs participating in inflammatory responses while performing defensive functions such as phagocytosis. Therefore, inducing the hybrid activation state of DAMs could contribute to inhibiting AD progression and improving plaque pathology [71].

Ultimately, microglia are activated into various functional phenotypes during the pathological processes associated with AD [53,72,73]. Notably, these phenotypic changes are being studied as crucial elements for understanding the pathological mechanisms involved in AD. In the early stages of AD, microglia are activated into an anti-inflammatory phenotype, secreting anti-inflammatory cytokines such as IL-4, IL-10, and IL-13, thereby performing neuroprotective functions. These microglia contribute to suppressing inflammation and promoting tissue repair, aiding in delaying the progression of the pathology. In contrast, during the later disease stages, microglia transition into an inflammatory phenotype, secreting proinflammatory cytokines such as IL-1α, IL-1β, TNF-α, and IL-6, as well as ROS. This leads to neuronal loss and accelerates the pathological progression of AD. Consequently, activated microglia induce neurotoxic effects and amplify inflammatory responses.

In conclusion, while microglia perform anti-inflammatory and neuroprotective roles in the early stages of AD, as the disease progresses to later stages, activated microglia induce inflammatory damage, promoting the pathological deterioration observed in AD.

### 2.2. Astrocytes

Astrocytes play a vital role in the CNS by supporting critical functions such as neural network development, synaptogenesis, the maintenance of the blood–brain barrier, and immune regulation [74,75]. Astrocytes can detect a wide range of signals, enabling them to respond to both endogenous and exogenous threats. Additionally, astrocytes sense endogenous DAMPs released from stressed or damaged neurons alongside exogenous PAMPs from invading pathogens or environmental toxins [76]. Astrocytes can also detect these harmful signals by expressing pattern recognition receptors (PRRs), such as TLRs and purinergic receptors, and activating the appropriate immune responses within the CNS [76,77,78].

The reactivity of astrocytes can be described through two main states characterized by morphological and molecular changes, analogous to the polarized states of microglia (M1 and M2). Early studies defined these two astrocyte activation states as A1 and A2 (Figure 1) [34,76]. A1 astrocytes are induced by activated microglia and exhibit neurotoxic activity, thereby losing their ability to phagocytose synaptosomes and myelin debris, which may be associated with the pathology of various neurological diseases [34,79]. Conversely, A2 astrocytes are characterized by anti-inflammatory functions and neuroprotective activities, which support neuronal protection and tissue repair in certain contexts [79,80]. However, additional reactive states have been identified, similar to microglia [81], indicating that the reactivity of astrocytes is not limited to just two states and can exhibit diverse changes depending on the type of disease or environmental context.

Glial fibrillary acidic protein (GFAP) is a key astrocyte filament protein and is an important marker for astrocyte activation and reactivity in the nervous system. Notably, when astrocytes are in a reactive state, the GFAP expression increases, reflecting their response to neuroinflammation and injury. Recent studies have shown that GFAP expression levels tend to be elevated in the blood and CSF of dementia patients, suggesting increased astrocyte activation and reactivity [82,83,84]. In this context, studies have shown that the morphology of astrocytes changes in mice with deleted *GFAP* and *vimentin* genes, and the number of amyloid plaques increases. This provides strong evidence that astrocytes closely interact with amyloid plaques [29,85]. Furthermore, astrocytes have been reported to bind and degrade Aβ directly around plaques in AD [86].

A recent review has indicated that the activation of astrocytes, similar to that of microglia, plays a significant role in developing neurodegenerative diseases such as AD [87]. Astrocytes exhibit various phenotypic changes in AD, which contribute to limiting pathological alterations associated with the growth of amyloid plaques [87]. These findings suggest that the activation of astrocytes is an important mechanism in the pathological progression of AD. Furthermore, astrocytes play a crucial role in cognitive functions, particularly in regulating synaptic plasticity and synaptic transmission [88,89,90]. These functions are associated with cognitive decline and disease progression. Indeed, activated astrocytes have been reported to initially provide neuroprotection by removing Aβ and supporting synaptic plasticity [86,91,92]. However, as the disease progresses and microglia density increases, astrocytes undergo a shift toward a more neurotoxic phenotype, leading to an increase in GABAergic signaling and a decrease in glutamatergic signaling, resulting in an imbalance in synaptic transmission in the hippocampus [93]. This emphasizes the dual role of astrocytes in both neuroprotection and the pathophysiological processes of neurodegeneration, underscoring the need for a nuanced understanding of astrocyte functions. Additionally, categorizing astrocyte reactivity into binary states, such as A1 and A2, represents an oversimplification that fails to capture the complex changes occurring across diverse cellular states and pathological contexts. According to the Brandebura review [94], therapeutic strategies targeting reactive astrocytes should focus on the diversity of astrocyte responses to neurodegeneration and the temporal dynamics of gene and protein expression changes during pathological progression. In this context, since the responses of microglia and astrocytes vary depending on time and environmental conditions, therapeutic strategies should carefully track these cellular changes and be customized to either promote the protective functions of microglia and astrocytes or alleviate the neurotoxic response according to the specific pathological state. This approach has also been emphasized in recent review papers [62,95]. To achieve this, an integrated approach from multiple perspectives is essential. For example, combining single-cell transcriptomic and proteomic analyses, morphological data, pharmacological data, or modulating signaling pathways can provide a more precise understanding of reactive microglia and astrocyte functions and inform the development of tailored therapeutic strategies.

## 3. Receptors Associated with Microglia in Neuroinflammation and AD

**Figure 2:**
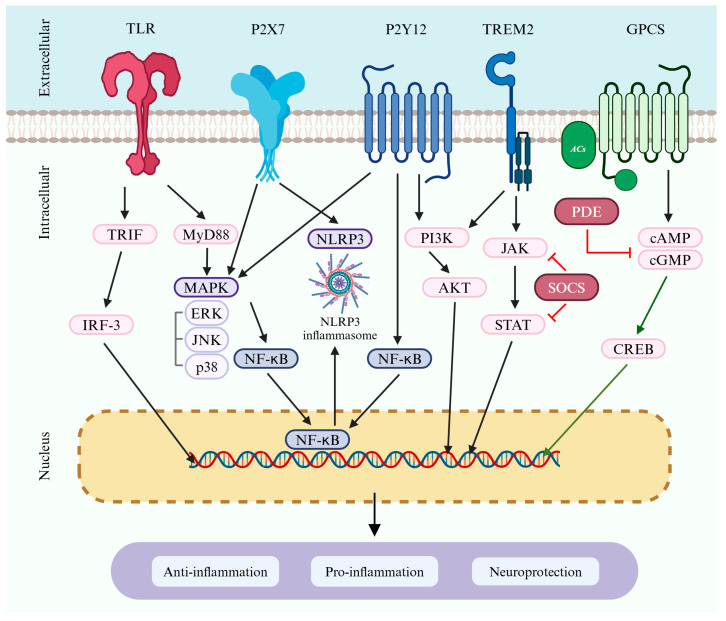
Microglial activation, key receptors, and signaling pathways in neuroinflammation. This diagram explains the major receptors and signaling pathways involved in microglia activation, highlighting their roles in neuroinflammation and neuroprotection. The key receptors include TLRs, purinergic receptors (P2X7 and P2Y12), and TREM2. TLRs recognize PAMPs and DAMPs that induce inflammatory responses. Purinergic receptors, P2X7 and P2Y12, respond to extracellular ATP, with P2X7 primarily promoting inflammasome activation and P2Y12 regulating microglial chemotaxis. Additionally, TREM2 recognizes damage-associated lipids and plays a critical role in modulating microglial responses to brain injury and neurodegenerative diseases. The major signaling pathways these receptors activate include MAPK, the NLRP3 inflammasome, PI3K/AKT, JAK/STAT, and cAMP/cGMP. Meanwhile, TLRs and purinergic receptors activate the MAPK and NF-κB pathways, promoting the expression of inflammatory genes. The NLRP3 inflammasome is activated by NF-κB and P2X7, inducing the release of inflammatory cytokines. The PI3K/AKT and JAK/STAT pathways are primarily activated by TREM2, with SOCS proteins acting as negative regulators to prevent excessive inflammation, thus modulating both proinflammatory and anti-inflammatory gene expression. Lastly, the cAMP/cGMP pathway promotes anti-inflammatory and neuroprotective responses through CREB activation. The network of these receptors and pathways regulates microglial activation, maintaining a balance between neuroinflammation and neuroprotection, and playing a crucial role in AD.

Microglia serve as key regulators of neuroinflammation and, thus, play a crucial role in detecting inflammatory responses and releasing key cytokines to modulate them. Not only do microglia initiate the inflammatory response, but they also significantly influence the subsequent actions of astrocytes. Therefore, in-depth research on microglia is essential, given their central role in various processes. Furthermore, understanding how receptors and signaling are associated with microglial activation function in neuroinflammation and AD is necessary for elucidating their roles and the underlying pathological mechanisms. This review briefly describes the receptors and signaling pathways associated with microglial activation (Figure 2) and summarizes studies targeting the regulation of these receptors and signaling pathways in neuroinflammation or AD models published after 2022 (Table 1).

### 3.1. Toll-like Receptors

The TLR signaling pathways play a crucial role in the immune system and perform key neuroinflammation functions. TLRs are primarily located on the cell membrane, where they recognize external pathogens and endogenous substances released during inflammatory responses, thus inducing immune reactions. TLRs also interact with Aβ [134,135,136].

The activation of TLRs occurs following stimulation by ligands through two main signaling pathways [137,138,139]. First, the MyD88-dependent pathway is activated when ligands stimulate TLRs, utilizing an adapter protein called MyD88. During this process, the IL-1 receptor-associated kinase (IRAK) and TNF receptor-associated factor 6 (TRAF6) are activated, promoting the activation of the NF-κB and mitogen-activated protein kinase (MAPK) pathways. The MyD88-dependent pathway increases the expression of inflammatory cytokines and chemokines, promoting the inflammatory response. Second, the TIR-domain-containing adapter-inducing interferon-β (TRIF)-dependent pathway involves TLR4 binding to TRIF, which activates interferon regulatory factor 3 (IRF3) and TANK-binding kinase 1 (TBK1), primarily contributing to the production of type I interferons. Most TLR subtypes display MyD88-dependent signaling, while TLR4 is unique because it utilizes both the MyD88-dependent and TRIF-dependent pathways [137].

TLRs are abundantly expressed in CNS microglia and astrocytes [140,141]. Thus, activating TLRs in these cells promotes their transition to an inflammatory phenotype. This process induces the release of cytokines and chemokines and enhances the immune response through antigen presentation on the cell surface [141]. Furthermore, TLR4 has been shown to significantly impact AD via TLR signaling [135,138].

TLR4 activation can have neuroprotective effects in the early stages. For example, in TLR4-deficient mice (Tlr(Lps-d)/Tlr(Lps-d)), Aβ uptake by microglia was impaired, and there was an increase in the diffuseness and deposition of Aβ compared to wild-type TLR4 mice. These data indicate that TLR4 plays a key role in Aβ clearance and neuroprotection [142]. Additionally, it was shown that the activation of TLR4 signaling in microglia at the early stage of β-amyloidosis reduces Aβ deposition and Aβ-mediated neurotoxicity [143]. Indeed, experiments with low-dose LPS treatment in transgenic mice overexpressing the human tau mutant (P301S) demonstrated that low-intensity TLR4 activation promotes autophagy, which significantly reduces Aβ levels [144]. This suggests that TLR4 may support neuroprotection through Aβ phagocytosis in the early stages of AD [144,145,146].

Alternatively, the long-term activation of TLR4 can lead to chronic inflammation, resulting in elevated inflammatory cytokine levels, which ultimately contribute to neurodegeneration and the progression of AD. Studies indicate that the long-term administration of LPS–TLR4 agonists induces neuroinflammation and cognitive deficits reminiscent of AD-related conditions [147,148]. This suggests that sustained activation of TLR4 may cause neuronal damage and functional decline, potentially leading to a pathological state similar to that seen in AD. Compounds designed to target TLR4 have demonstrated an ability to reduce cognitive impairments and enhance AD-like symptoms in animal models [138]. For example, alpha-linolenic acid (ALA), an omega-3 polyunsaturated fatty acid, was shown to improve amyloid generation and memory deficits by inhibiting TLR4 and its downstream targets, including neuroinflammation, apoptosis, amyloid precursor protein (APP), and beta-secretase 1 (BACE-1) [149]. Using the TLR4 inhibitor TAK-242, the study investigated how TLR4 inhibition promotes the polarization of microglia to the M2 phenotype, providing neuroprotective effects in AD [150]. The TLR4 antagonist IAXO-101 was shown to reduce neuroinflammation and improve memory in mice overexpressing the *APOE4* gene, which is associated with sporadic AD [151]. In summary, therapeutic approaches targeting TLRs have the potential to enhance our understanding of the pathological mechanisms involved in AD and contribute to the development of effective treatments. Therefore, it is important to investigate the mechanisms involved in TLR4 action further to explore ways to achieve both neuroprotection in the early stages of inflammation and its long-term suppression.

In terms of clinical results, PTI-125 is a known TLR4-targeting agent. PTI-125 is a small molecule drug that binds to filamin A (FLNA) to block Aβ42 binding to the α7 nicotinic acetylcholine receptor (α7nAChR) while also inhibiting Aβ42 from binding to TLR4, thereby reducing inflammation and neuronal damage. The results from phase 2 clinical trials indicated positive changes in several biomarkers, including the CSF (total tau/Aβ42) ratio, with reported decreases in tau protein and Aβ levels [152]. To date, no further research has been conducted. As of 2024, the clinical agent used to target TLR was CpG 1018, an exclusive immunoadjuvant composed of nucleotides that mimic bacterial DNA, which functions by binding to TLR9 and activating the innate immune system. This treatment was used as an adjuvant in the hepatitis B vaccine approved in 2017 and was included in COVID-19 vaccines [153]. Currently, a phase 1 clinical trial is underway, and preclinical research has shown that CpG ODNs promote the phagocytic activity of microglia [154]. CpG ODN treatment has also demonstrated the ability to modulate microglial function, reducing amyloid deposition and preventing cognitive deficits in the Tg2576 AD model and 3xTg AD mice [155,156].

### 3.2. Purinergic Receptors

Purinergic receptors play a crucial role in regulating the state and function of microglia under various conditions, serving as a key pathway in the modulation of neuroinflammation. Microglia express various P2X and P2Y receptors, as well as P1 subtypes. ATP or other purinergic signaling molecules can activate and be released extracellularly due to pathological events or in response to physiological brain activity [157,158,159,160]. The various subtypes of these purinergic receptors are closely linked to the dynamic phenotypes of microglia, allowing them to adapt and respond effectively to their environment. As microglial activation progresses, these cells undergo phenotypic changes mediated by the interactions of various purinergic receptors, enabling them to perform functions such as phagocytosis, anti-inflammatory actions, or the release of inflammatory mediators [161,162].

When the P2Y12 receptor is activated in response to ATP released from damaged cells, it induces morphological changes and mediates the migration of microglia to the site of damaged or overactive neurons [162,163]. Therefore, the P2Y12 receptor influences the dynamic phenotypes of microglia and activates the PI3K/AKT, NF-κB, and MAPK pathways, thereby contributing to the activation of proinflammatory pathways such as the NLRP3 inflammasome, which enhances inflammation in microglia [164,165]. In addition to P2Y12, ATP-gated P2X7 receptors are abundantly present in microglia [166]. Indeed, the P2X7 receptor (P2X7R) regulates key intracellular pathways such as MyD88/NF-κB, PI3K/AKT/mTOR, and the NLRP3 inflammasome, leading to the release of inflammatory cytokines, production of reactive oxygen and nitrogen species, and induction of cell apoptosis [167,168,169,170]. Furthermore, the sustained activation of P2X7 induces changes in intracellular ions through pore formation, and these changes promote neuroinflammation and neurodegeneration, playing a crucial role in the progression of inflammatory and neurodegenerative diseases [170,171,172,173]. P2X7R is known to be activated only at high ATP concentrations in the millimolar range [174]. However, P2X7R is also described as a phagocytic receptor, even without an external ATP agonist, and plays an important role in innate phagocytosis in the brain [175]. This suggests that P2X7R is crucially involved in various microglial functions beyond merely triggering inflammation. Notably, while the activation of P2X7R can lead to neuroinflammation and neurodegeneration, P2X7R activation can also promote phagocytic activity even without external ATP, highlighting the complex role of P2X7R. This implies that P2X7R significantly influences the activation state of microglia and pathological changes. 

The P2X7 receptor was discovered in microglia nearly 20 years ago and has since become one of the most studied elements in the purinergic signaling system, particularly in relation to various CNS diseases, including AD [176,177]. The expression of P2X7 receptors increases in many neurological disorders such as AD, and pharmacological blockade or genetic deletion of P2X7 has been shown to have neuroprotective effects [177]. Aβ induces increases in the intracellular calcium ion concentration (Ca^2+^), ATP release, and IL-1β accumulation in microglia from normal mice compared to P2X7-deficient mice, highlighting the essential role of the P2X7 receptor in Aβ-mediated microglial activation [178]. Furthermore, several studies have confirmed that P2X7 receptors are upregulated in AD models [179,180]. Inhibition of the P2X7R has been shown to regulate pathways involving glycogen synthase kinase 3β (GSK-3β) and secretases [181], thereby modulating chemokine release induced by Aβ and reducing Aβ generation and accumulation [182]. P2X7 has also been found to play an important role in neurodegeneration caused by tau proteins. Meanwhile, the expression of P2X7 receptors increased in a tauopathy mouse model [183,184]. Comparatively, microglia activation and inflammatory mediators were reduced in P2X7-deficient tau mice. Furthermore, P2X7 deficiency has been described to restore memory and synaptic plasticity in tau mice [184]. Subsequently, the role of activated P2X7 receptors in impairing the function of the ubiquitin–proteasome system (UPS) in microglia, thereby inhibiting the degradation of tau protein, is being studied [102].

The P2X7R plays a critical role in the microglial inflammatory response to neurodegeneration, making it a significant therapeutic target, particularly in relation to AD and tau pathology. Thus, understanding and regulating the function of the P2X7R is essential for developing therapeutic approaches for neurodegenerative diseases.

### 3.3. TREM2

TREM2 is an important receptor almost exclusively expressed in brain microglia and is essential for regulating immune responses. TREM2 detects the microenvironment of the CNS and is involved in modulating inflammation and phagocytic activity in microglia [185,186,187,188,189]. TREM is a transmembrane glycoprotein, and the extracellular domain of TREM2 can bind to Aβ, LPS, and various apolipoproteins. These ligands activate TREM2 signaling [186,190,191,192]. TREM2 mediates intracellular signaling and various microglial physiological functions by binding to the adaptor protein DNAX-activation protein 12 (DAP12) and regulating multiple signaling pathways. TREM2 mediates phagocytosis through the SYK/PI3K/AKT pathway and suppresses inflammation by regulating the PI3K/NF-κB and Janus kinase/signal transducer and activator of transcription (JAK/STAT) pathways [186,193]. In the early stages, DAMs are activated independently of TREM2, but microglia progress to a TREM2-dependent stage. At this stage, the TREM2 receptor is essential for the complete activation of the cells into DAMs, enabling microglia to perform more complex functions such as phagocytosis [71].

Research using mice with *TREM2* gene mutations has shown that the decrease in TREM2 expression reduces microglial density and decreases the expression of anti-inflammatory genes [194]. In particular, the anti-inflammatory response induced by IL-4 is weakened due to the reduction in TREM2 expression [194]. Additionally, TREM2 knockdown inhibits the phagocytic activity of microglia towards apoptotic neurons and increases the transcription of TNF-α and NO synthase-2, thereby promoting inflammatory responses. In contrast, TREM2 overexpression enhances phagocytic activity and reduces the proinflammatory responses of microglia, facilitating the effective clearance of dead neurons [189]. This indicates that TREM2 regulates the inflammatory response in the brain and plays a critical role in inducing anti-inflammatory responses for neuroprotection. Due to these characteristics, TREM2 has emerged as an important target for research related to the progression of neuroinflammation in AD [186].

Recent studies using genome sequencing followed by imputation have identified a rare missense mutation in the *TREM2* gene that increases the risk of AD [5,195]. These findings suggest that *TREM2* mutations affect the function of microglia, thereby elevating the risk of AD [5,185]. Many studies and reviews indicate that TREM2 plays a crucial role in the pathology of AD [5,189,195,196,197]. TREM2 was upregulated in microglia in the APPswe/PS1dE9 mouse model in response to increased levels of amyloid-β (Aβ1–42). Subsequent experiments manipulating TREM2 expression have shown that it promotes the phagocytosis of Aβ and suppresses Aβ-induced proinflammatory responses, thereby improving neuropathology and enhancing spatial cognitive function in AD [198]. Additionally, while there was no direct effect on Aβ plaque deposition in the TREM2^+/−^ PPPS1–21 mouse model, a decrease in microglia associated with Aβ plaques was observed [199]. Furthermore, TREM2 helps microglia respond to Aβ accumulation by sensing damaged lipids related to neurodegeneration. Research has also indicated that TREM2 mutations prevent microglia from clustering around Aβ plaques and lead to cell death [200]. This suggests that TREM2 in microglia is a critical regulator in AD progression, facilitating an appropriate response to Aβ plaques and playing a vital role in their clearance. Therefore, TREM2 could be an important therapeutic target in AD.

While TREM2 plays a crucial role in regulating the function and state of microglia in AD, its function can vary depending on specific interactions and environmental contexts. Studies in APP/PS1 and SOD1 mouse models have shown that TREM2 can shift microglia from a homeostatic state to a neurodegenerative state through APOE signaling [201]. This suggests that TREM2 may promote anti-inflammatory responses; however, in certain situations, it can also facilitate pathological changes associated with neurodegenerative states via interactions with APOE. In conclusion, mutations in TREM2 or its reduced expression can significantly impact the functional changes in microglia and the regulation of inflammatory responses. However, understanding the mechanisms through which TREM2 and APOE interact to regulate the function and state of microglia in neurodegenerative diseases is crucial.

Antibodies targeting TREM2 have been used to enhance the survival, proliferation, and phagocytic activity of microglia, offering a potential therapeutic strategy for AD [202]. AL002 is a monoclonal antibody targeting the TREM2 receptor on microglia and is currently in phase 2 clinical trials. In published preclinical studies, acute administration of AL002 in TREM2-modified models induced metabolic activation and proliferation of microglia, while long-term treatment reduced filamentous plaques and neurite dystrophy, positively influencing behavior and alleviating the inflammatory response of microglia [203]. Additionally, AL002 is a safe and well-tolerated drug previously employed in a phase 1 study involving healthy volunteers, where it dose-dependently decreased soluble TREM2 levels in the CSF and increased concentrations of biomarkers associated with TREM2 activation, such as CSF1R, SPP1, and IL1RN, thus demonstrating microglial activity [204,205]. These findings suggest that using AL002 to activate microglia through the TREM2 pathway may help regulate neuroinflammation and reduce pathological changes, potentially slowing or preventing AD progression.

## 4. Signaling Associated with Microglia in Neuroinflammation and AD

### 4.1. JAK/STAT Signaling Pathway

The JAK/STAT signaling pathway plays a crucial role in cellular functions, regulating immune system activity, tissue repair, inflammation, and apoptosis [206,207]. Over 70 cytokines utilize the JAK/STAT pathway [208]. JAK binds to cytokine receptors and phosphorylates the tyrosine amino acids in the receptors, which recruit STAT proteins. Phosphorylated STAT proteins form dimers and translocate to the nucleus, where they regulate the expression of specific genes [207]. Among these genes are those that produce suppressors of cytokine signaling (SOCS) proteins. These SOCS proteins (such as SOCS1, SOCS2, and SOCS3) act as negative feedback regulators, inhibiting further phosphorylation of JAK and modulating the inflammatory response. Cytokines rapidly induce SOCS proteins, creating a negative feedback mechanism that inhibits the JAK/STAT signaling pathway [209]. The JAK2/STAT3 pathway plays a crucial role in microglia polarization, primarily inducing polarization toward the proinflammatory M1 phenotype. Indeed, following JAK2 activation, STAT3 becomes phosphorylated and translocates to the nucleus, promoting the transcription of various inflammation-related genes. In this process, the expressions of cytokines such as TNF-α and IL-1β increase, enhancing the inflammatory response [210,211]. Research indicates that external stimuli, such as paraquat (PQ), activate the JAK2/STAT3 pathway in microglia, promoting a transition to the M1 phenotype, which increases the expression of inflammatory cytokines and markers [212]. Using a microglia-specific *STAT3* knockout mouse model, it was confirmed that STAT3 deficiency induces the transition of microglia from M1 to M2 polarization, inhibits proinflammatory responses, and promotes anti-inflammatory responses, thereby alleviating early neuronal damage [213]. Additionally, SOCS proteins act as negative regulators of this pathway. In LPS-activated microglia, a decrease in SOCS-1 leads to an increase in M1-like cells that promote the production of inflammatory cytokines, while M2-like cells decrease, disrupting the anti-inflammatory changes in microglia. This indicates that when SOCS-1 expression is reduced, the JAK2/STAT3 pathway is activated, promoting polarization toward the microglia M1-like phenotype [214]. Moreover, the JAK2/STAT3 pathway was found to regulate microglia/macrophage polarization during cerebral ischemia/reperfusion injury [215]. Contrary to the previous findings, STAT3 deficiency failed to promote M2 polarization and instead increased the expression of proinflammatory cytokines, thereby exacerbating neurological damage [215]. Although conflicting results exist in the literature, a comprehensive analysis suggests that the JAK2/STAT3 pathway plays a critical role in the polarization transition of microglia and inflammation under certain conditions. Therefore, future studies should aim to clarify the regulatory mechanisms of this pathway. 

Recent GWASs have analyzed changes in the JAK/STAT signaling pathway and its association with inflammatory processes in AD, with the data suggesting that this pathway may be a potential therapeutic target for AD [216]. Indeed, numerous studies have demonstrated that the JAK/STAT3 pathway is crucial in promoting neuroinflammation and pathological changes in AD [217,218]. For example, STAT3 phosphorylation is critical for the secretion of cytokines associated with neuroinflammation, an early pathological phenomenon in AD. Therefore, STAT3 inhibition significantly reduces inflammation-induced neuroinflammation and Aβ42 and BACE1 levels [218,219].

It was also previously found that astrocytes transition to a reactive state through STAT3 in AD [217,220]. Here, STAT3 phosphorylation occurs upon oligomeric Aβ treatment, leading to increased astrocyte reactivity, which causes impairments in learning and memory [217,220]. This reactive state may increase the release of inflammatory cytokines, leading to neuronal cell death and further neurodegeneration.

In experiments using AD models, it was reported that inhibiting the JAK/STAT/SOCS signaling pathways suppresses the expression of inflammatory cytokines, thus aiding in the recovery of cognitive deficits [221]. Additionally, an increase in SOCS3 expression was shown to play a crucial role in altering the microglia phenotype through the JAK/STAT signaling pathway, thereby regulating neuroinflammation in an AD model [222]. However, some studies have observed that the levels of p-STAT3 in hippocampal neurons decrease with age in AD mouse models and patients and that the inactivation of JAK2/STAT3 by Aβ is associated with memory impairment related to AD [223,224]. Thus, the JAK/STAT3 signaling pathway is crucial in neuroinflammation and is associated with AD. Furthermore, activation of the JAK/STAT3 signaling pathway promotes the secretion of inflammatory cytokines and induces the reactive state of microglia, affecting memory. However, some studies have published conflicting results, suggesting that activation of the JAK/STAT3 signaling pathway may exacerbate neuroinflammation. This indicates that the regulation of this pathway may have different effects depending on the context in which it is activated. Therefore, a more cautious and multifaceted approach is required when considering the role of the JAK/STAT3 pathway and its therapeutic potential.

Baricitinib is a selective JAK2/STAT3 inhibitor that suppresses the immune response and reduces inflammation by interfering with cytokine signaling. Baricitinib was first approved by the FDA in 2018 to treat rheumatoid arthritis and is being explored for various other disorders; for example, baricitinib is currently in clinical trials for AD treatment. In preclinical studies, baricitinib has been shown to alleviate the abnormal activation of the JAK2/STAT3 signaling pathway induced by ovariectomy and D-galactose, reduce neuroinflammation, decrease astrocyte hyperactivity, and lower Aβ levels, thereby improving spatial learning and memory [225].

### 4.2. NOD-like Receptor Signaling

NLRs are crucial innate immune system components, acting as cytosolic PRRs that detect various intracellular signals. These receptors play a significant role in microglia activation, an important aspect of neuroinflammation, particularly impacting neurodegenerative diseases such as AD [226,227]. Among the many inflammasomes reported, the NLRP3 inflammasome is the most extensively studied in relation to neuroinflammation and degenerative diseases [228]. The NLRP3 inflammasome complex comprises the NLRP3 sensor protein, an adapter protein known as the apoptosis-associated speck-like protein containing a CARD (ASC), and cysteine protease procaspase-1; this protein complex is important in the innate immune system [229].

NLRP3 activation specific to microglia is known to contribute to the pathological progression of AD [227]. Activation of the NLRP3 inflammasome has been observed specifically in plaque-associated microglia of APP/PS1 mice [230]. Furthermore, the NLRP3 inflammasome acts as an Aβ sensor, regulating the phagocytosis of Aβ and the synthesis of proinflammatory and neurotoxic substances, ultimately playing a critical role in the recruitment of microglia to exogenous Aβ in the brain [231]. In Nlrp3^−/−^ or Casp1^−/−^ mouse models, there was a reduction in spatial memory loss, suppression of caspase-1 and interleukin-1β activation in the brain, and enhanced clearance of amyloid-β [230]. Additionally, MCC950, a reversible inhibitor that maintains NLRP3 in an inactive state [232], has been shown to inhibit LPS- and amyloid-β-induced caspase-1 activation in microglia, reduce IL-1β secretion, and enhance cognitive function by decreasing amyloid-β accumulation while inhibiting the inflammasome and microglial activation in the APP/PS1 model [233].

The NLRP3 inflammasome is also implicated in tau pathology [227]. A significant reduction in hyperphosphorylated tau levels was observed in the hippocampus, CA1 cell body region, and dentate gyrus in control tauopathy models compared to tauopathy models in which the components of the NLRP3 inflammasome were disrupted (Tau22/Asc^−/−^ and Tau22/Nlrp3^−/−^ mice); these outcomes also led to the recovery of spatial memory deficits. This process occurs sequentially, with Aβ fibers activating the NLRP3 inflammasome, a crucial process in inducing tau pathology [234]. NLRP3 deficiency reduced tau hyperphosphorylation and aggregation and inhibited tau propagation, decreasing tau pathology in the hippocampus and cortex. Additionally, NLRP3 deficiency significantly reduced hippocampal atrophy and attenuated the progression of tau-related neurodegeneration [235].

In summary, the NLRP3 inflammasome is a key element that induces and exacerbates AD and tau pathologies, suggesting that targeting NLRP3 with therapeutic strategies has the potential to slow the progression of AD and improve cognitive function.

### 4.3. MAPK Signaling

The MAPK signaling pathway is crucial in transmitting external signals to induce various intracellular responses, such as cell proliferation and differentiation. This pathway consists of several key components that facilitate a series of protein activations, ultimately regulating gene expression and cellular behavior [236]. The MAPK pathway is typically initiated when signaling molecules bind to receptors on the cell surface, which triggers the activation of MAPK kinase kinase (MAPKKK), followed by a cascade of protein kinases. Subsequently, MAPK kinase (MAPKK) is activated, initiating effector kinases mediating various cellular responses [236,237].

The MAPK pathways can be broadly classified into three main routes: First, the ERK pathway responds to growth factors and cytokines, promoting cell proliferation, differentiation, and development, and plays a critical role in cell growth and survival. Second, the c-Jun N-terminal kinase (JNK) pathway is activated by stress and cytokines, regulating apoptosis and stress responses to help eliminate damaged cells or adapt to stress. Third, the p38 pathway responds to inflammatory stimuli, TGF-β, and stress, regulating inflammatory responses, cell death, and development, and plays an important role in immune responses and adaptation to stress [238]. According to research, the MAPK signaling pathway plays a crucial role in the polarization and inflammatory response of microglia. Naringenin and dexmedetomidine target the JNK and ERK1/2 pathways, respectively, inducing the transition from M1 to M2 polarization and exhibiting anti-inflammatory effects [239,240]. Additionally, lysophosphatidic acid increases the inflammatory properties of microglia through a MAPK-dependent pathway. Meanwhile, the reduced production of inflammatory cytokines upon treatment with MAPK antagonists suggests that the MAPK signaling pathway plays an important role in LPA-induced inflammation [241]. Thus, the MAPK signaling pathway is central to the functions and polarization of microglia, indicating the potential of using various compounds to regulate inflammatory responses. The polarization and inflammatory response of microglia are essential for maintaining brain homeostasis and appropriately responding to pathological conditions, making the MAPK pathway an important area of research in neuroinflammation and neurodegenerative diseases [237,242].

The MAPK signaling pathway in microglia plays a crucial role in studying the pathological mechanisms involved in AD. The MAPK signaling pathway mediates neuroinflammatory responses and is closely associated with the onset of AD. Aβ increases M1-type microglia and decreases M2-type microglia, contributing to the promotion of inflammatory responses by activating the ERK, JNK, and p38 pathways [243]. Acutely isolated microglia showed prominent ERK phosphorylation in the 5xFAD mouse model, closely associated with regulating AD-related genes (*Trem2* and *Tyrobp*) and several AD risk genes. Additionally, an analysis of the proteome in human brain tissue post-mortem indicated that increased ERK1 and ERK2 expressions were positively correlated with neuropathological grading, providing evidence of ERK signaling pathway activation in AD. These findings highlight the importance of ERK phosphorylation within microglia as a crucial regulator in the pathogenesis of AD [244]. However, interesting research findings suggest that the JNK and p38–MAPK pathways activated by sAPPα are major mediators of neuroinflammation, indicating that while ERK is involved in microglial activation, it is not the primary regulator of the observed inflammatory responses [245]. Nevertheless, it remains possible that the ERK signaling pathway modulates microglial inflammatory responses and contributes to the pathological progression of AD. Recently, research has shown that aucubin targets the ERK/FOS signaling pathway to inhibit the abnormal activation of microglia, thereby alleviating AD-like pathological features such as Aβ plaque deposition, neuronal damage, and inflammation due to astrocyte hyperactivation in the brains of APP/PS1 mice [122]. These findings suggest that regulating the ERK pathway could be vital for treating AD.

JNK is known to activate β- and γ-secretases, which are involved in cleaving the APP in neuronal cells [246,247]. Furthermore, there is evidence that Aβ peptides can promote JNK activation [248,249]. This suggests that the accumulation of Aβ activates JNK, which in turn promotes the generation of Aβ, forming a reinforcing relationship that plays a significant role in the generation and progression of NFTs [250]. Recent studies have shown that magnoflorine significantly inhibits the expression of phosphorylated JNK in an AD model, improving cognitive deficits. This reduced the concentration of Aβ plaques and phosphorylated tau protein (p-tau) levels, contributing to the inhibition of NFT formation, which are pathological features of AD. Additionally, the decrease in Iba1 expression indicates that microglial activation was suppressed, leading to reduced inflammatory responses [251].

The p38–MAPK pathway has been studied more actively than other MAPK pathways in the context of AD and neurodegenerative diseases. Indeed, p38 primarily induces inflammatory responses in microglia and astrocytes and promotes microglial activation through Aβ42 [252,253,254,255]. In addition, p38 inhibition alleviates the toxic effects of tau in microglia, enhances tau phagocytosis and processing capabilities, and promotes an increase in the number of lysosomes and the removal of tau aggregates [256].

Studies utilizing p38α/β–MAPK inhibitors have shown downregulation of phosphorylated p38–MAPK levels in AD models, reduced Aβ deposition, and improved spatial learning and memory loss. Interestingly, while the levels of IL-1β and TNF-α decreased, indicating a reduction in inflammatory status, no significant changes or increases in the number of microglia, activation markers, or phagocytic receptor expression have been reported [257,258]. In contrast, reports indicate that the selective degradation of p38–MAPK can reduce microglial activation [125]. This suggests that the inhibition of p38–MAPK may not consistently reduce microglial activation across experimental conditions. Therefore, while the role of p38–MAPK and its effects on microglial activation need to be clarified through further research, in AD, the activation of p38–MAPK plays a crucial role in microglial inflammatory responses and disease progression, making it a promising therapeutic target.

Presently, MW150, a p38–MAPK kinase inhibitor that targets mild to moderate AD, is in phase 2 clinical trials to assess its therapeutic effects. The inhibition of p38α–MAPK has shown significant potential for treating AD in preclinical tests. In preclinical studies, MW150 demonstrated the ability to inhibit hippocampal-dependent associative and spatial memory deficits in AD-related APP/PS1 knock-in mouse models, effectively modulating neuroinflammatory responses by reducing inflammatory cytokines [257,259].

### 4.4. PI3K/AKT Signaling

The PI3K/AKT signaling pathway regulates the activation and function of microglia, influencing both proinflammatory and anti-inflammatory responses. The PI3K/AKT signaling pathway is considered a crucial modulator of microglial function in response to external triggers, including inflammatory cytokines and LPS [260,261]. Microglial cells activated by external stimuli activate PI3K, which promotes the activation of AKT. The activation of AKT leads to its translocation to the nucleus, where it regulates various downstream proteins. These targets include key proteins in cell survival, apoptosis, protein synthesis, and cell growth. Thus, focusing on the inflammatory response, molecules such as NF-κB and mTOR are regulated by AKT activation and play crucial roles in the immune response and inflammatory process [261,262].

On the other hand, the PI3K/AKT signaling pathway can induce an anti-inflammatory response in microglia, whereby the activation of AKT may contribute to neuroprotection. Microglia stimulated by important receptors for inflammation regulation, such as TREM2 or the transcription factor IRF3, activate the PI3K/AKT signaling pathway, promoting M2 polarization [263,264,265]. In other words, activating the PI3K/AKT pathway increases the expression of anti-inflammatory factors, such as IL-10 and IL-4, while suppressing the expression of proinflammatory genes, thereby performing an anti-inflammatory role [263,264]. Additionally, mGluR5-positive allosteric modulators have been shown to activate the PI3K/AKT signaling pathway in microglia, mediating anti-inflammatory effects through the GSK-3β and cAMP response element-binding protein (CREB) signaling pathways [266]. Therefore, the PI3K/AKT signaling pathway can exhibit either proinflammatory or anti-inflammatory effects depending on the environmental context, indicating that the factors regulating this pathway could be explored as promising therapeutic targets for inflammatory diseases.

Additionally, the PI3K/AKT signaling pathway plays a crucial role in the mechanisms underlying the onset of AD and other neurodegenerative disorders [267,268,269]. This pathway is essential for neuronal survival and function, and the accumulation of Aβ oligomers disrupts the propagation of the PI3K/AKT pathway, leading to cell death and cognitive decline. Dysfunction in the PI3K/AKT pathway can accelerate the neuronal apoptosis observed in AD, whereas overexpression of AKT has been demonstrated to significantly reduce apoptosis induced by Aβ1–42 [270,271]. Additionally, Aβ oligomers can directly interfere with the PI3K/AKT pathway, leading to the activation of GSK-3β, which plays a critical role in regulating the production of inflammatory molecules in microglial cells. Activation of this kinase enhances cytokine and chemokine production by stimulating multiple signaling pathways, including JNK, STAT3/5, and NF-κB. Moreover, GSK-3β promotes β-catenin degradation, reducing its inhibition of NF-κB to promote inflammation further. These mechanisms contribute to an increased production of proinflammatory mediators, potentially worsening inflammatory diseases [272]. In an AD model, inhibition of the PI3K/AKT pathway suppressed the secretion of inflammatory mediators and cytokines, reduced the proliferation of inflammatory cells in microglia and astrocytes, decreased Aβ deposition, and improved cognitive function [129]. Similarly, the inhibition of the PI3K p110δ isoform has been shown to positively affect key AD symptoms, such as plaque formation, neuroinflammation, and cognitive decline, further supporting the potential of targeting the PI3K pathway in the therapeutic intervention of AD [273].

In both normal and early disease stages, the autophagy system is essential for removing Aβ oligomers and hyperphosphorylated tau [274]. Meanwhile, research has suggested that the autophagic process utilizing the PI3K/AKT/mTOR pathway could be leveraged for AD therapy [275,276]. Research on astragalin has presented neuroprotective effects by activating autophagy by inhibiting the PI3K/AKT/mTOR pathway in APP/PS1 mice [275]. Another study describes how arctigenin regulates the AKT/mTOR signaling pathway to activate autophagy, promoting the production and clearance of Aβ. This mechanism emphasizes the relationship between the effects of arctigenin and cognitive improvement [276]. The dual PI3K/mTOR inhibitor, BEZ (NVP-BEZ235, BEZ), was reported to improve memory deficits and reduce microglial activation in the T41 AD mouse model. However, it was noted that BEZ does not affect Aβ levels or neuronal damage markers such as NeuN and GFAP [277]. Therefore, the dual inhibition of PI3K/mTOR using BEZ does not affect Aβ deposition or neuronal survival; however, BEZ could regulate inflammatory responses, and microglial activation positively impacted memory and cognitive function improvement in AD. This finding is noteworthy and suggests that neuroinflammation is critical in AD treatment.

Therefore, while PI3K inhibitors may be a promising therapeutic option for alleviating neuroinflammation and cognitive decline, their efficacy may be limited against key pathological mechanisms such as Aβ accumulation and neuronal damage. Therefore, to maximize the therapeutic effects of PI3K inhibitors, combination therapy with other agents that effectively target Aβ plaque accumulation and neuronal damage may be necessary. This approach could provide a more comprehensive strategy to address the diverse pathological mechanisms of AD.

### 4.5. Cyclic Nucleotide Signaling

Cyclic nucleotide signaling is an important mechanism that regulates various physiological processes in the CNS and has significant implications for neuroinflammation and AD. These intracellular signaling molecules modulate multiple responses, including neuronal function, neuroplasticity, memory function, inflammation, cell proliferation, and apoptosis, making them key targets in the research of neurodegenerative diseases [278].

Cyclic adenosine monophosphate (cAMP) is synthesized from ATP by adenylate cyclase (AC) and is regulated by G-protein coupled receptors (GPCRs). When a ligand activates a GPCR, the Gα subunit either activates or inhibits AC, thereby regulating cAMP production. The generated cAMP activates various downstream signaling molecules, including protein kinase A (PKA), CNGC, and Epac. Notably, PKA phosphorylates CREB, promoting the transcription of genes critical in neuroplasticity and memory formation. The activity of PKA is terminated through a negative feedback mechanism involving phosphodiesterase (PDE), which degrades cAMP [279].

Cyclic guanosine monophosphate (cGMP) is generated from GTP by guanylate cyclase (GC), which exists in two forms: particulate GC and soluble GC (sGC). Notably, sGC is activated by nitric oxide (NO), which is produced in response to increased calcium levels; activated sGC converts GTP to cGMP, which then regulates synaptic transmission through protein kinase G (PKG) and is also involved in the indirect phosphorylation of CREB [279].

Over the past several decades, research has indicated that cyclic nucleotide signaling possesses immunosuppressive and anti-inflammatory effects, which is partly attributed to the ability of cyclic nucleotide signals to interfere with the function of the inflammatory transcription factor NF-κB [280,281,282,283]. These data demonstrate that cyclic nucleotide signaling reduces inflammatory signaling in various cells, including microglia. Meanwhile, cAMP is a crucial intracellular regulator of microglial homeostasis, and microglia become activated when the cAMP level decreases due to inflammatory signals. For example, inflammatory stimuli such as TNF-α activate inflammatory-related proteins in microglia, such as iNOS, COX-2, ERK1/2, and NF-κB–p65, triggering an inflammatory response. In contrast, high levels of cAMP inhibit these inflammatory pathways and reduce the expression of inflammatory mediators [281]. This suggests that cAMP plays a crucial role as an important regulator of the inflammatory response.

The cyclic nucleotide signaling pathway affects the motility and polarized phenotypic transition of microglia. The cAMP level plays a crucial role in the transition of microglia from a proinflammatory phenotype (M1) to an anti-inflammatory phenotype (M2) [284]. Studies have shown that combining cAMP and IL-4 promotes the transition from M1 to M2 through cAMP [284]. Additionally, an increase in cAMP facilitates nanoscale filopodial sensing, while a decrease in cAMP leads to the extension of larger processes, inducing broader immune surveillance [285]. Alternatively, cGMP is an important molecule that regulates the motility of microglia. Research indicates that when LPS stimulates BV-2 cells, NO is produced, which binds to sGC to increase cGMP production. This increase in cGMP promotes the motility of microglia, while inhibition of cGMP production decreases their motility. Therefore, cGMP plays a positive role in regulating the movement of microglia [286].

For these reasons, the cyclic nucleotide signaling pathway is gaining attention as a target for inflammation suppression and treating neurological diseases. One of the key enzymes in this pathway, PDE, plays a role in terminating signals by degrading cAMP and cGMP. Subsequently, PDE inhibitors are being researched as potential therapeutics for inhibiting microglial activation. PDEs are a large family of enzymes that hydrolyze 3′-phosphodiester bonds in the cAMP and cGMP signaling pathways, generating 5′-cyclic nucleotides, thus terminating cyclic nucleotide signaling [287]. Therefore, administering PDE inhibitors could play an important role in inhibiting microglial activation, and the therapeutic potential of PDE inhibitors is being explored in various neuroinflammatory models. For example, the PDE-4 inhibitor rolipram has been shown to enhance memory in streptozotocin-treated and naturally aged mouse models, alleviating neuroinflammation through reduced acetylcholinesterase activity, increased oxidative stress markers, and decreased myeloperoxidase activity [288]. In Tg APP/PS1 mice, cognitive decline, neuroinflammatory responses, and impaired cGMP signaling have been observed; however, the use of the PDE5 inhibitor sildenafil restored memory deficits and signaling impairments, which were blocked by the injection of the cGMP-dependent protein kinase inhibitor Rp-8-Br-PET-cGMPS. Consequently, PDE5 inhibitors may recover cognitive deficits in Tg APP/PS1 mice through modulating PKG/p-CREB signaling and anti-inflammatory responses [289].

Particularly, PDE inhibitors have been reported to activate the CREB signaling pathway, contributing to restoring synaptic function and improving AD symptoms. Indeed, learning and memory impairments were induced in mice microinfused with Aβ1–42, and administering the PDE2 inhibitor Bay 60-7550 improved these impairments further; meanwhile, the effects were blocked by PKG and PKA inhibitors, as well as by inhibiting p-CREB and BDNF [290]. Additionally, studies using double transgenic mice with APP (AA substitution K670N and M671L) and presenilin-1 (AA substitution M146V) showed that the PDE4 inhibitor rolipram improved long-term potentiation (LTP) and contextual learning deficits. This protective effect was confirmed to be associated with activating the PKA/CREB signaling pathway [291]. Moreover, various studies have consistently shown that PDE inhibitors, by activating the AC/cAMP/PKA or NO/cGMP/PKG signaling pathways, increase cAMP and cGMP levels in the brain, elevate CREB levels, enhance synaptic transmission, and reduce cognitive deficits [278,292].

The small molecule inhibitor AR1001, which targets PDE-5, is currently in phase 3 clinical trials for AD following preclinical studies that indicated that AR1001 could increase cGMP, improve memory and learning abilities, reduce amyloid production, and alleviate neuroinflammation [13].

The CNS consists of various cell types, including neurons, microglia, astrocytes, and oligodendrocytes; each performs distinct physiological functions through specific receptors and signaling modules. While some receptors and signaling modules are specialized for particular cell types, others, such as those involved in immune signaling and/or synapse regulation, can function across multiple cell types. For instance, a recent review [293] reported that cAMP plays different roles depending on the cell type. In neurons, it promotes neuroplasticity, survival, and signaling, while in microglia and astrocytes, it primarily exerts anti-inflammatory effects. Additionally, cAMP plays an important role in oligodendrocytes, supporting myelination and cell differentiation. However, the downstream effector of cAMP, EPAC, reduces neuroinflammation in microglia but induces neuronal apoptosis through the p38–MAPK and PI3K/AKT/GSK3β/JNK pathways in neurons. This variability suggests that cAMP regulates diverse physiological functions through distinct signaling pathways in different cell types. Therefore, focusing on modulating specific downstream pathways could be a promising therapeutic strategy. Since cAMP is activated in multiple cell types, it can play a key role in regulating the interactions between microglia, astrocytes, and neurons. Furthermore, integrated therapeutic strategies could be developed by simultaneously modulating the cAMP pathway across different cell types. For example, therapies could be designed to induce anti-inflammatory effects in microglia, promote survival in neurons, and enhance myelination in oligodendrocytes. Nevertheless, research focusing on microglia-specific targeting is critical because neuroinflammation is a major pathological feature of neurodegenerative diseases, and microglia play a central role in mediating this inflammatory response. Indeed, studies have reported successful targeted drug delivery using nanoparticles or liposomes conjugated with ligands or specific antibodies that target microglia receptors, enabling precise drug delivery [294,295,296]. Therefore, future research should focus on developing integrated therapeutic strategies that regulate specific downstream pathways or modulate multiple cell types simultaneously to achieve microglia-specific targeting while minimizing off-target effects. Additionally, approaches that precisely regulate microglia activation using ligands or specific antibodies targeting microglia receptors will be necessary.

## 5. Conclusions

The development of therapies targeting neuroinflammation in AD is increasingly recognized as a crucial area of research in the search for effective treatments. Neuroinflammation plays a significant role in the onset and progression of AD; thus, targeting this process with anti-inflammatory therapies has the potential to alleviate symptoms and slow disease progression. Comprehensive reviews of various anti-inflammatory drug candidates currently in clinical trials have also highlighted the important possibilities of focusing on neuroinflammation in AD treatment, suggesting new directions for understanding the complex pathological mechanisms of the disease.

Microglia and astrocytes are typically described in a classical binary manner, but this approach risks oversimplifying the complex aspects observed in AD. Rather than being compartmentalized into specific states, these cells exist in a dynamic continuum of activation states that change in response to various environmental signals and pathological conditions. This diversity encompasses several intermediate phenotypes formed in response to environmental cues, significantly impacting the functional roles of microglia and astrocytes. Moreover, the ability of these cells to flexibly transition between active states adds complexity to our understanding of their roles in AD, suggesting that appropriate activation in specific pathological contexts may be a critical factor in regulating disease progression.

Microglia play a vital role in neurodegenerative diseases such as AD, and the activation state of microglia profoundly impacts disease progression and recovery. While previous research has primarily focused on inhibiting the abnormal activation of microglia, recent findings emphasize that microglia can remain in an activated state while still performing neuroprotective and clearing functions. The signaling pathways related to microglial activation significantly impact both neuroprotection and inflammation, making it essential to regulate multiple signaling pathways in a balanced manner for effective management. In the early stages of AD, enhancing the protective functions of microglia is crucial, while in the later stages, strategies to regulate chronic inflammation become necessary. Hence, it is important to continuously monitor the activation state and cellular changes in microglia, as well as fine-tune the signaling pathways to manage their activation state carefully. Future AD treatments will focus on regulating microglial activation and inflammatory responses in an integrative manner while enhancing the protective functions of microglia. This approach can extend beyond AD and serve as an effective therapeutic strategy for other neurodegenerative diseases where neuroinflammation plays a significant role.

Overall, understanding the complex functions of microglia and finely regulating their activation states may be the key to maximizing the effectiveness of AD treatments. Ultimately, this could lead to the development of more effective therapies, offering new perspectives on neurodegenerative diseases and improving the quality of life of patients. Such research is expected to serve as a foundational resource for establishing future preventive and therapeutic strategies for AD.

## Figures and Tables

**Table 1:** Regulation of receptors and pathways associated with microglial activation in neuroinflammation and Alzheimer’s disease models. Downregulation (↓) and Upregulation (↑).

Pathway	Model	Target	Refs.
TLR2/MyD88	BV-2 cell line	TLR2↓, MyD88↓, NLRP3↓, IL-1β↓, TNF-α↓, IL-6↓, IL-8↓, Iba-1↓	[96]
TLR4/NF-κB	APP/PS1 AD mouse model	ROS↓, IL-1β↓, TNF-α↓, IL-6↓, TUNEL-positive cells↓, APOE↑, TREM2↑, APOE↑, TLR4↓, nuclear NF-κB↓, cytoplasmic NF-κB↓, Iba-1↓	[97]
TLR4/MyD88	OVX/D-Gal + scopolamine-induced rat model	Histopathological score↓, intact neuron count↓, Aβ42↓, p-tau↓, NF-κB↓, NLRP3↓, caspase-1↓, IL-1β↓, IL-18↓, TLR4↓, MyD88↓, TRAF-6↓, TAK-1↓, p-JNK↓, CD86-positive microglia↓, CD163-positive microglia↑	[98]
TLR3	APP/PS1 AD mouse model	Aβ↓, Iba-1↓, GFAP↓, CD68↓, NeuN+ cell↑, CD206↓, CD68↓, CD16/32↓, NF-κB↓, IL-1β↓, TNF-α↓, IL-4↑	[99]
TLR4/NF-κB	Aβ–ICV-induced mouse AD model, BV-2 cell line	Survival neurons↑, apoptosis index↓, GFAP↓, Iba-1↓, NF-κB↓, TLR4↓, IκBα↓, NeuN+ cell↑, ROS↓, iNOS↓, COX-2↓, TNF-α↓, IL-1β↓, IL-6↓, apoptotic cells↓	[100]
TLR and ubiquitin–proteasome	TgF344 AD rat model	Aβ↓, tau PHF↓, amoeboid/ramified microglia↓	[101]
P2X7R and PI3K/Akt/GSK3/Nrf2	Human AD patients, P301S mice, N2a cell line	p-GSK3↑, Nrf2- and FK2-positive intracellular aggregates↓	[102]
A1R/A2AR and BDNF	STZ-ICV-induced AD rat model	A1R↑, A2AR↓, TrkB↑, BDNF↑, BUchE↓, cholesterol↓, IL-4↑, IL-10↑	[103]
TREM2/NF-κB	SAMP8 mice, BV-2 cell line	iNOS↓, Arg-1↑, IL-10↑, PU.1↑, TREM2↑, p-NF-κB↓, TNF-α↓, IL-6↓, IL-1β↓, p-IKKβ↓	[104]
TREM2/TLR4/NF-κB	BV-2 cell line	TREM2↑, TNF-α↓, IL-1β↓, IL-6↓, IL-10↑, CD11b↓, iNOS↓, Arg-1↑	[105]
TREM2/PI3K/AKT	Dysfunction mouse model, BV-2 cell line	TREM2↑, p-NR2B↑, BDNF↑, IL-1β↓, IL-6↓, p-PI3K↑, p-AKT↑, Iba-1↓, iNOS↓	[106]
JAK/STAT	LPS-induced mouse, BV-2 cell line	IL-1β↓, IL-6↓, TNF-α↓, iNOS↓, JAK↓, STAT↓, Nrf2↑, HO-1↑	[107]
NFκB/IL6/STAT3	APP/PS1 AD mouse model, HT-22, BV2 cell line	NeuN↑, Aβ↓, p-STAT3↑↓, iNOS↓, p65↓, IL-6↓, IL-1β↓, IL-6↓, TNF-α↓	[108]
JAK2/STAT3	Scopolamine-induced BALB/c mouse, LPS- and Aβ-treated BV-2 cell line	AchE↓, ChAT↓, p-JAK2↓, p-STAT3, iNOS↓, Arg-1↑, IL-1β↓, IL-6↓, TNF-α↓, IL-12↓, IL-4↑, IL-10↑	[109]
JAK/STAT	LPS-treated BV-2 cell line, primary microglial	NO↓, IL-6↓, TNF-α↓, COX-2↓, iNOS↓, p-ERK↑, p-JNK↓, p-p38↓, IL-11↓, JAK2↓, Tyk2↓, STAT1↓, STAT3↓, neurite length↑	[110]
JAK1–STAT6	APP/PS1 AD mouse model, BV-2 cell line	p-JAK1↑, p-STAT6↑, SOCS3↑, CD206↑, Iba-1↑	[111]
Autophagy–NLRP3 inflammasome, PINK1/Parkin	APP/PS1 AD mouse model	AchE↓, MDA↓, IL-1β↓, MCP-1↓, IL-6↓, IL-18↓, TNF-α↓, SOD↑, Aβ plaques↓, tau↓, LC3B↑, p62↓, PINK1↑, parkin↑, beclin1↑, LC3II/LC3I↑, NLRP3↓, ASC↓, cleaved caspase-1↓	[112]
SIRT6/NLRP3	Aβ–ICV-induced AD rat model	SIRT6↑, H3K9Ac↓, p-NF-κB↓, IκBα↓, NLRP3↓, ASC↓, caspase-1↓, cleaved caspase-1↓, GSDMD↓, IL-18↓, Iba-1↓, GFAP↓, COX-2↓, TNF-α↓, ROS↓, NO↓, IFN-γ↓, IL-1β↓, IL-6↓, ROS↓, MDA↓, IL-4↑, IL-10↑, TGF-β↑, SOD↑, GSH↑	[113]
NLRP3	HMC-3 human microglia cell line, scopolamine-induced mouse model	JC-1↓, NLRP3↓, NF-κB↓, vimentin↓, ROS↓, AchE↓, BChE↓, MDA↓, SOD↑, catalase↑	[114]
NLRP3/GSDMD	STZ-ICV-induced mouse model (C57BL/6J)	NLRP3↓, caspase-1↓, IL-1β↓, GSDMD↓, caspase-3↓	[115]
NLRP3/caspase-1	Aβ-treated BV-2 cell line, APP/PS1 AD mouse model	NLRP3↓, ASC↓, caspase-1↓, IL-1β↓, IL-18↓, ROS↓	[116]
NLRP3	3xTg AD mouse model	p-tau↓, Aβ↓, p-GSK3β↑, NeuN↑, Bcl-2↑, Bax↓, Iba-1↓, GFAP↓, IL-1β↓, TNF-α↓, IL-6↓, IL-18↓, p-NF-κB, IKKβ↓, NLRP3↓, ASC↓, caspase-1↓, GSDMD-N↓	[117]
NLRP3	LPS- + IFN-γ-treated BV-2 cell line	TNF-α↓, IL-6↓, IL-1β↓, NLRP3↓, ASC↓, caspase-1↓, CD11b↓, ROS↓	[118]
TLR4/MAPK	APP/PS1 AD mouse model	Aβ↓, MDA↓, SYP↑, PSD95↑, IL-1β↓, IL-6↓, TNF-α↓, COX-2↓, CD14↓, Fos↓, Igf2↓, MAPK13↓, CCL3↓, CCL4↓, TREM2↑, TLR4↓, MyD88↓, JNK↓, p38↓, Iba-1↓, CD86↓, iNOS↓, ARG1↑, CD206↑, IL-1β, IL-6, TNF-α↓CD86, iNOS↓, ARG1, TREM2↑, TLR4, MyD88, JNK, p38↓	[119]
MAPK	LPS-treated BV-2 cell line	NO↓, iNOS↓, COX-2↓, p-p38↓, p-JNK↓, p-ERK↓, NLRP3↓, ASC↓, cleaved-caspase-1↓, IL-1β↓, pro-IL-1β↑, PGE_2_↓, TNF-α↓, IL-6↓	[120]
MAPK/NF-κB	LPS-treated BV-2 cell line	NO↓, PGE2↓, IL-6↓, TNF-α↓, MCP-1↓, COX-2↓, p-ERK↓, p-JNK↓, p-p38↓, p-IκB↓, p-p65↓	[121]
MAPK/ERK	APP/PS1 AD mouse model, LPS-treated BV-2 cell line	Aβ plaques↓, Iba-1↓, GFAP↓, p-ERK1/2↓, c-FOS↓, FOSB↓, FOSL1↓, ROS↓, iNOS↓, NO↓, IL-1β↓, IL-6↓, TNF-α↓, COX-2↓	[122]
MAPK/ERK	3xTg AD mouse model	Aβ25-35↓, TNF-α, ↓ IL-1β↓, IL-6↓NeuN↑, CD31↑, GFAP↓, VCAM-1↓, ICAM-1↓, p-ERK↓, p-MAPK↓, Aβ1–42↓, p-tau↓	[123]
p38/NLRP3 inflammasome	3xTg AD mouse model, BV-2 cell line	Aβ plaque↓, PSD95↑, synaptophysin↑, TNF-α↓, IL-1β↓, IL-6↓, IL-4↑, IL-10↑, Iba-1↓, CD16↓, Arg1↑, NLRP3↓, ASC↓, caspase-1↓, IL-18↓, FIS1↓, DRP1↓, OPA1↑, MFN2↑, TFAM↑, NRF1↑, p-p38↓, JC-1↓	[124]
MAPK/p38	LPS-treated BV-2 cell line, 5XFAD AD mouse model	IL-6↓, IL-1β↓, IL-12↓, TNF-α↓, p-p38↓, Aβ↓, Iba-1↓, GFAP↓, p-tau↓	[125]
PI3K/AKT	APP/PS1 AD mouse model	iNOS↓, CD11b↓, TREM2↑, TMEM119↑, Iba-1↓, Aβ↓, p-AKT↑, p-PI3K↓, p-tau↓, IL-1β↓, NF-κB↓, iNOS↓	[126]
PI3K/AKT/mTOR	AlCl3-triggered AD rat model	Aβ↓, PI3K↑, AKT↑, mTOR↑, GSK3β↑, BACE-1 ↓, p-tau↓, iNOS↓, NF-κB↓	[127]
PI3K/AKT	LPS-ICV-induced mouse	iNOS ↓, pro-IL-1β↓, NO↓, GSK3β↑, SOD↑, GPX1↑, CREB↑	[128]
PI3K/AKT	5xFAD AD mouse model	Thioflavin↓, Iba-1↓, GFAP↓, NF-κB↓, p-IκBα↓, p-AKT↓	[129]
PI3K/AKT/GSK3β	APP/PS1 AD mouse model	Iiba-1↓, Aβ↓, p-AKT↑, p-PI3K↑, p-tau↓, GSK3β↑	[130]
PDE4	Rotenone, oral injection, rat model	DA level↑, PDE4 activity↓, pCREB↑, PKA↑, PP-1↓, BDNF↑, NGF↑, NF-κB↓, GSH↑, IL-6↓, TNF-α↓	[131]
PDE3	C57BL/6 (23 months)	NOR↓, GFAP↓, Iba-1↓	[132]
PDE2	MCAO C57BL/6	PDE2↓, cerebral edema↓, TNF-α↓, IL-6↓, IL-1β↓, MCP-1↓, NF-κB↓, PKA↑, Iba-1↓	[133]

## Data Availability

Not applicable.
